# Jottings

**Published:** 1910-03-19

**Authors:** 


### Jottings.

The Princess of Wales, attended by Lady Mary Trefusis, visited the Mildmay Mission Hospital at Bethnal Green last Saturday.

At the annual meeting of the Cancer Hospital, Fulham, a vote of thanks for services rendered to the charity by the Press was carried unanimously.

Ledbury Cottage Hospital (Hereford), in spite of money spent upon painting and renovating the building, has a balance on the year of £43.

The Prince of Wales's Hospital, Tottenham, owing to an accident, has lost part at least of the £50 worth of radium with which it was presented some time ago.

H.R.H. Princess Henry of Prussia visited the German Hospital, Dalston, on Friday, March 11, and was shown round the institution by the Chairman and members of the House Committee.

The wage-earners of Southport have increased the weekly contributions to the Southport Infirmary by 125 per cent., that is from £122 to £275. The date for the infirmary ball has been fixed for April 6.

In reply to a request for a subsidy owing to the increased number of patients sent by the Medical Officer of the Bath Education Authority, the Bath Eye Infirmary has received a reply that the Authority cannot subsidise charitable institutions.

The Princess of Wales visited the Friedenheim Hospital in Upper Avenue Road, Swiss Cottage, on March 10.

The out-patients for the past year at the Scarborough Hospital and Dispensary have decreased considerably, but as the death-rate for the town has fallen from 719 in 1907 to 540 in 1909, this is a tribute to the improving healthiness of Scarborough.

The Million Penny Fund instituted by the matron of the Bromley Cottage Hospital is making good progress. It is proposed largely to extend this excellent little hospital, a sum of £2,500 being needed to undertake the necessary alterations and improvements.

The Chelmsford and Essex Hospital and Dispensary, though there was a deficit on the year's working, was able to show a balance of £61 on the Building Fund. The buildings were finished during the past year, and £6,893 had been subscribed for the Fund.

The extension of the west wing, the enlarged children's ward, and new balcony of the Cheltenham General Hospital have now been formally opened by Viscount St. Aldwyn, an outline of the scheme being given by Colonel Croker-King, the president of the hospital.

The Royal Liverpool Country Hospital for Children, the well-known feature of which is the lengthy stay it allows if necessary to complete cure, has made proposals to the Liverpool Education Committee to provide instruction for such children, which are expected to fructify shortly.

In order to combat the growing expenditure and the continual falling-off of subscriptions to the Dorset County Hospital, Canon Rowland Hill, a vice-patron, proposed to raise to £2 2s. an in-patient ticket, and secondly to reduce to one month the period for which such a ticket could last.

Littlehampton feels the urgent need for more adequate hospital accommodation. It has accordingly been decided to enlarge and improve the local institution, and a building fund has been started with that object. The fund now stands at £450, and an anonymous supporter has promised £100 provided a further sum of £400 is raised.

Mr. Bertram Abel Smith, chairman of the Nottingham General Dispensary, stated at the annual meeting that every subscriber knew that the money he gave was not wasted, as the committee consisted of business men who gave up some time each week to the details of management. Legacies last year amounted to £944.

The Prince and Princess of Wales, the Duke and Duchess of Connaught, Prince and Princess Christian, Princess Louise (Duchess of Argyll), and Princess Henry of Battenberg have given their patronage to the annual ball in aid of the Italian Hospital, Queen Square, to be held at the Royal Institute of Painters in Water Colours, Piccadilly, on Tuesday, May 24.

Mr. George Ferguson, chairman of the directors of the Royal Samaritan Hospital for Women, Glasgow, at the annual meeting, said it was the first time they were able to close the year with a credit balance, owing to the Dick bequest of £10,000 and to the success of the fancy-dress ball which realised £1,500. At the present moment there is £7,000 to the credit of the hospital.

The Dundee Victoria Hospital for Incurables has, after 10 years' work, proved to the working classes of the town that to send a man to such an institution is not to proclaim him a doomed man, but to guarantee that his infirmity shall be rendered as slight as possible. Lord Provost Urquhart, who presided at the recent annual meeting, joined other speakers in congratulating the citizens on their progressive change of view.

